# Serum and Antibodies of Glaucoma Patients Lead to Changes in the Proteome, Especially Cell Regulatory Proteins, in Retinal Cells

**DOI:** 10.1371/journal.pone.0046910

**Published:** 2012-10-11

**Authors:** Katharina Bell, Sebastian Funke, Norbert Pfeiffer, Franz H. Grus

**Affiliations:** Experimental Ophthalmology, Department of Ophthalmology, Medical Center of the Johannes Gutenberg University, Mainz, Germany; Massachusetts Eye & Ear Infirmary, Harvard Medical School, United States of America

## Abstract

**Purpose:**

Previous studies show significantly specifically changed autoantibody reactions against retinal antigens in the serum of glaucoma and ocular hypertension (OHT) patients in comparison to healthy people. As pathogenesis of glaucoma still is unknown the aim of this study was to analyze if the serum and antibodies of glaucoma patients interact with neuroretinal cells.

**Methods:**

R28 cells were incubated with serum of patients suffering from primary open angle glaucoma (POAG), normal tension glaucoma (NTG) or OHT, POAG serum after antibody removal and serum from healthy people for 48 h under a normal or an elevated pressure of 15000 Pa (112 mmHg). RGC5 cells were additionally incubated with POAG antibodies under a normal pressure. Protein profiles of the R28 cells were measured with Seldi-Tof-MS, protein identification was performed with Maldi-TofTof-MS. Protein analysis of the RGC5 cells was performed with ESI-Orbitrap MS. Statistical analysis including multivariate statistics, variance component analysis as well as calculating Mahalanobis distances was performed.

**Results:**

Highly significant changes of the complex protein profiles after incubation with glaucoma and OHT serum in comparison to healthy serum were detected, showing specific changes in the cells (e.g. Protein at 9192 Da (p<0.001)). The variance component analysis showed an effect of the serum of 59% on the cells. The pressure had an effect of 11% on the cells. Antibody removal led to significantly changed cell reactions (p<0.03). Furthermore, the incubation with POAG serum and its antibodies led to pro-apoptotic changes of proteins in the cells.

**Conclusions:**

These studies show that the serum and the antibodies of glaucoma patients significantly change protein expressions involved in cell regulatory processes in neuroretinal cells. These could lead to a higher vulnerability of retinal cells towards stress factors such as an elevated IOP and eventually could lead to an increased apoptosis of the cells as in glaucoma.

## Introduction

Glaucoma, a group of diseases leading to loss of retinal ganglion cells (rgc) with still unknown pathogenesis, is a leading cause for blindness worldwide, as the estimated number of affected people counting nearly 7 million shows [1]. The most common form of glaucoma, the primary open angle glaucoma (POAG), is accompanied by an elevated intraocular pressure (IOP). But approximately 30% of the patients don't show an elevated IOP (normal tension glaucoma (NTG)) [2]. Additionally 4–7% of people over the age of 40 have ocular hypertension (OHT) [3] but per year only 1% develop glaucoma [4], [5]. Therefore, although still a major risk factor, the presumption that an elevated IOP is the only cause for glaucoma is long out-dated. Glaucoma can be added to the long list of neurodegenerative diseases with mostly unknown pathogenesis, conditions characterized by progressive nervous system dysfunction and often accompanied by the atrophy of the affected central or peripheral nervous system structures [6].

As in other neurodegenerative diseases, such as amyotrophic lateral sclerosis, Alzheimer's and Parkinson disease, glaucoma leads to the apoptotic loss of one specific neuron population, the rgc [7]. An atrophy of central structures such as the lateral geniculate nucleus [8] can also be found. Keeping the number of people affected in mind, glaucoma can be contemplated as one of the most common neurodegenerative diseases [9]. Many possible aspects to the pathway of destruction of the rgc other than an apoptosis [10], [11] through elevated IOP are being discussed, most importantly an elevated nitric oxide level [12], a T-cell mediated process [13] or an autoimmune process [14], all including an involvement of intracellular cascades leading to cell death [15], [16], which could be possibly caused by a crucial serum factor present in glaucoma patients. Changes in the antibody spectra towards retinal or optic nerve antigens in glaucoma patients (POAG and NTG) but also OHT patients in comparison to healthy people [17], [18] were demonstrated and give a hint to this crucial serum factor. The existence of autoantibodies against retinal or optic nerve head antigens also in healthy people is part of the so called natural autoimmunity [19], which has been demonstrated in many other studies [20].

Considering antibody (Ab) expressions changes and studies showing rgc apoptosis after treatment with an elevated hydrostatic pressure [11], our aim was to determine the effect of serum and Abs from glaucoma patients and different pressure levels on R28 and RGC5 cells, not only by analyzing the viability, but also taking a deeper look at the processes of the cell during different treatments by measuring their protein expression profiles. A cell cultural approach with the established neuroretinal cells R28 [21] [provided as a gift by G M. Seigel; Ross Eye Institute, University of Buffalo] and the established RGC5 cell line [22], [23] [provided as a gift by N. Agarwal; Fort Worth, Texas USA] was performed, incubating the cells with serum from POAG, NTG and OHT patients, serum from healthy people and POAG serum after Ab removal under a normal or elevated pressure. Furthermore the cells were treated with the Ab's from POAG patients.

We hypothesize that the serum of glaucoma patients can lead to changes in cell regulatory processes of the cells which make the cells more vulnerable to stress factors and eventually can lead to apoptosis of the cells. Furthermore we believe that the antibodies in the serum of glaucoma patients play a major role in this effect.

## Materials and Methods

### Ethics Statement

The studies were performed in accordance with the Declaration of Helsinki on medical research involving human subjects. Informed consent from all participants giving serum in for this study was obtained in a written form. The ethics committee of the Landesärztekammer Rheinland-Pfalz approved this study, approval number: No: 817.219.07(5754).

### Cell culture

The neuroretinal cell line R28 [provided as a gift by G M. Seigel; Ross Eye Institute, University of Buffalo] and RGC5 cells [provided as a gift by N. Agarwal; Fort Worth, Texas USA] were used. The R28 cells were cultured in Dulbecco's modified Eagles Medium (DMEM) containing 10% Fetal Bovine Serum (FBS; Cambrex Bioscience, Verviers, Belgien), 5 mg/ml Gentamicine- Glutamine Solution (Sigma-Aldrich GmbH, Steinheim), 10% MEM Vitamins (100× (Invitrogen)) and 10% MEM non essential amino acids (100× (Invitrogen)). RGC5 cells were cultured in DMEM containing 10% FBS, 1% Penicillin/Streptomycin (Sigma-Aldrich GmbH) and 4% L-Alanyl-L-Glutamine (Biochrom). Both were grown in a humidified atmosphere of 95% air and 5% CO_2_ at 37°C.

### Experimental setup

R28 cells were incubated with 10% non heat inactivated serum from POAG, NTG or OHT patients or healthy people for 48 h. One experiment was conducted using POAG and healthy serum (n each group = 9). In a further experiment additionally NTG and OHT serum was used (n each group = 12). An experiment using POAG, healthy and POAG serum after Ab removal with protein G beads was conducted (n each group = 4). The cells were incubated at a normal pressure or an elevated pressure of 15000 Pa in culture dishes with an 82.7 mm inner diameter. 250 000 cells were used. After 48 h, cell lysis was performed (Buffer: Urea 9.5 M, Chaps 2%, DTT 1%+proteinase inhibitor mix (P 1860 (Sigma-Aldrich)) with an ultrasonic pulse echo instrument (Labsonic®M (Sartorius). Protein concentration was determined with the method of Lowry [24].

A titration curve using different concentrations of healthy or POAG serum was performed. R28 cells were incubated under an elevated and normal pressure with different serum concentrations (2.5, 5, 7.5 and 10%; n in each group = 4).

Protein profiles were measured with Seldi-TOF MS. Protein identification was performed with Maldi-TOFTOF-MS.

Additionally, RGC5 cells were incubated with 5% FBS+ 5% healthy or 5% POAG serum or+the ABs of 5% POAG serum. After cell lysis, a SDS gel was performed with the pooled samples. The lanes were divided into 15 pieces and digested with trypsin, to measure the peptide profile with capillary LC-ESI-MSMS.

The viability of R28 cells after treatment with different serum types (POAG, healthy, bovine) and under different pressure conditions was measured with WST-1 test (Roche, Mannheim). The cells were grown in 96 well plates and treated as described above (n of each group = 20). After 48 h WST-1 reagent was added and the cells were measured using an Elisa plate reader at 450 nm.

### Serum samples

The serum was collected from patients suffering from POAG, NTG or OHT according to the classification of the guidelines of the European glaucoma society as well as from healthy volunteers after given informed consent. Furthermore patients suffering from autoimmune diseases or other neurodegenerative diseases were excluded from the study. In general no systemic diseases were dominantly represented in one of the experimental groups and therefore a correlation between the measured effects and a systemic disease can be excluded.

### Protein profiling with Seldi- TOF-MS

An acetone precipitation of 150 µg protein was undertaken. 8 times the amount of −80°C acetone was added and incubated on ice (30 min). The samples were centrifuged (14000 rpm, 4°C, 30 min). 2 µl of the sample was spotted on the protein chips for Seldi-Tof-MS using weak cationic exchanger (CM10) or reversed-phase surface (H50) chips. A surface-enhanced laser desorption/ionisation time-of-flight mass spectrometer (PBS-II SELDI-TOF (Ciphergen Biosystems Inc., Fremont) was used. The samples were covered with sinapic acid matrix (20 mg Sinapic acid, 750 µl acetonitrile (ACN), 750 µl H_2_O-HPLC, 15 µl Trifluoroacetic acid (TFA)). Measurements were performed according to the manufacturer's protocol.

### Protein identification with Maldi-TOFTOF-MS

**M**atrix-**A**ssisted-**L**aser-**D**esorption/**I**onization based **m**ass-**s**pectrometry (Ultraflex MALDI TOF/TOF (Bruker Daltonik GmbH, Bremen)) was used to identify the significantly changed proteins. After protein separation via SDS-Page (Invitrogen), a tryptic digestion of the bands including the significantly changed proteins was performed. In order to detect whether the specific protein was included in the protein band of the gel, an elution of the proteins was performed and measured with Seldi-TOF-MS. Only after detecting the wanted protein in the measurement, the tryptic digestion was performed. 1 µl of the digested proteins was loaded onto a Maldi anchor target using 2×0.5 µl cinnamic-acid matrix (17 mg α-Cyano-4-hydroxycinnamic acid, 4.8 mL HPLC H_2_O, 5 mL ACN, 200 µl TFA). Measurements were performed according to the manufacturer's protocol. Protein identification was performed using the mascot search engine using SwissProt database.

### Protein profiling with capillary LC-ESI-MSMS

RGC5 cells were analyzed with capillary LC-ESI-MSMS using a C-18 pre-column (30 mm×0.5 mm) and a C18 analytical column (150 mm×0.5 mm, both Thermo Scientific,). A Rheos Allegro HPLC Pump (Thermo Scientific) was the solvent delivery system. The pump flow rate was 200 µl/min, and reduced to a column flow of 10 µl/min (M-472 graduated microsplit valve (Upchurch, Scientific, USA)). With two running buffers (A(98%H_2_O, 1.94% ACN, 0,06% methanol, 0.05% TFA)+B(95% ACN, 3% methanol, 2% H_2_O, 0.05% TFA) a linear gradient of 80 min was performed (0–47 min: 0–100%B, 47–49 min: 100% B, 49–58: 100% -0% B, 58–80 min: 0%B). Mass spectra were obtained using an LTQ OrbitrapXL.

### Statistical analysis

A list of peak clusters measured with Seldi-TOF MS was generated and exported to a statistical analysis program (Statistica, ver. 8.0; Statsoft, Tulsa, OK). Multivariate discriminant analysis based on combinations of multiple protein peaks was calculated. A variance component and mixed model ANOVA was calculated to determine the influence of the dependent (serum, pressure) and independent variables on the protein profiles. The Mahalanobis distances were calculated to show changes after Ab removal.

The data from the capillary LC-ESI-MSMS analysis was used for quantification of the identified proteins in the different samples, after normalizing the intensity of the proteins using the total ion current (TIC). A comparison to the cells incubated with healthy serum was performed.

### Antibody depletion

The Ab's for the first study were removed using magnetic protein G beads (Dynabeads®Protein G; Dynal Biotech). 20 µl beads were used to purify 35 µl serum. They were washed (2×600 µl NaAc, pH5 2 min, 1×5 min), added to the POAG serum and incubated at 12°C on an orbital shaker for 6 h. The beads with Abs were discarded and the remaining serum was used for cell incubation. The antibodies were washed from the beads and measured with Seldi-Tof-MS to prove the removal from the serum or at least a large amount of Ab depletion (see Figure S1 for Seldi-spectrum).

For the following study with Ab depletion, the Abs were removed with the Nab Protein G Spin Kit (Thermo scientific), which removes IgG from samples, e.g. serum. The Abs can be released from the columns. Ab removal from the serum was confirmed by measuring the Abs in a SDS gel (see Figure S2) showing Ab bands and their fractions [25].

## Results

### Glaucoma serum changes cell proteins

**Figure 1:**
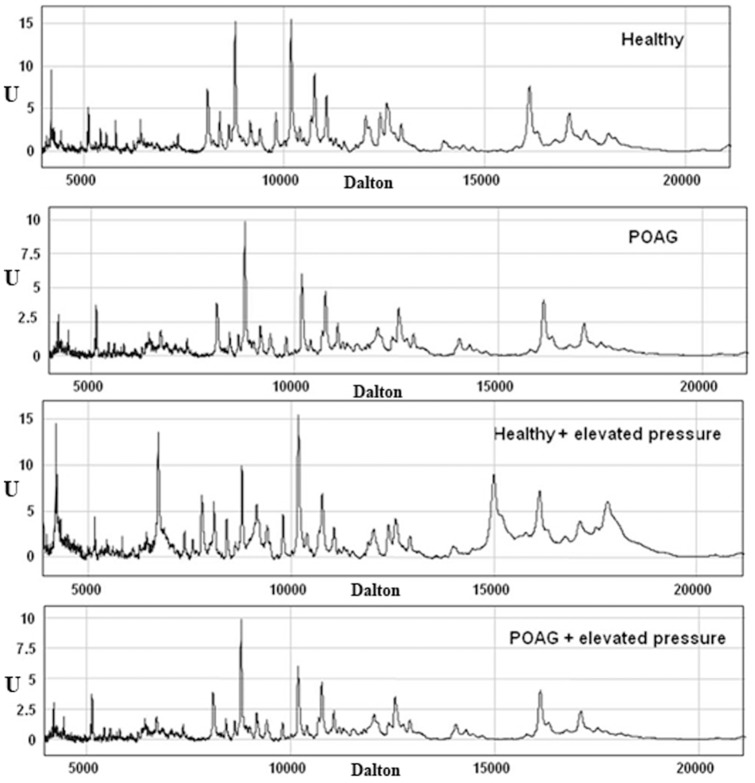
Complex protein profiles in every experimental group. Figure 1 shows examples of protein profiles measured with Seldi-TOF MS. Protein profiles from different experimental groups are shown. The X axis shows the molecular weight in Dalton and the Y axis the intensity of the protein in the sample. Differences between the experimental groups can be seen.

The very complex protein and peptide profiles of the R28 cells showed approximately 400 different protein clusters ranging from 3078 Dalton (Da) to 180 kDa. Some sample measurements can be seen in Figure 1 showing Seldi-TOF MS measurements from cells of different experimental groups. The analysis of discriminance showed several highly significantly different regulated proteins, when comparing cells incubated with POAG serum to cells incubated with healthy serum, The significantly changed proteins are listed in Table S1. The table shows the molecular weight of the proteins as well as the calculated p- values. Some of the differently expressed proteins of R28 cells incubated with POAG serum could successfully be identified using Maldi-TofTof-MS e.g. the protein at 9192 Da (Histone4) and the protein at 12390 Da (Profilin) (also listed in Table S1).

**Figure 2:**
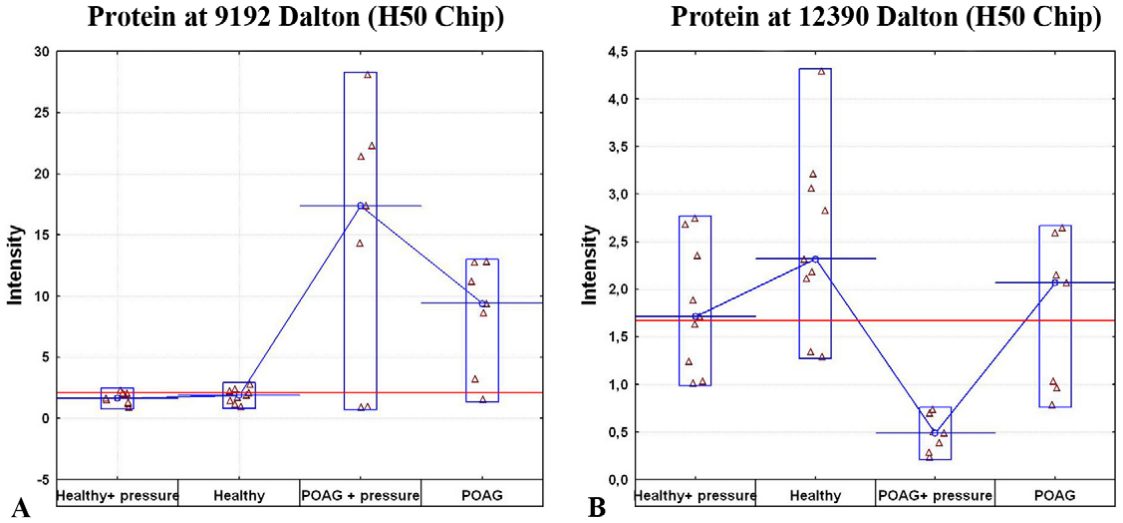
Significantly differently regulated proteins in cells incubated with glaucoma serum. Figure 2A and 2B show several variability plots of the calculated significantly changed proteins with the molecular weights of 9192 and 12390 Dalton. The X axis represents the different experimental groups and the Y axis shows the intensity of the protein measured by Seldi-Tof-MS. Each triangle in a plot represents one sample of the specific group. The protein at 9192 Da is up-regulated in those cells incubated with POAG serum as well as the cells incubated with POAG serum and an elevated pressure, whereas the protein at 12390 Da is down-regulated in those cells incubated with POAG and an elevated pressure.

Figure 2 A and B shows several of the significantly differently regulated proteins. The proteins at 9192 Da (p = 0.000058) and at12390 Da (p = 0.000086) are shown. To also detect overall changes in the protein profiles of the cells, the Mahalanobis distances were calculated, showing not only significant changes of single proteins but also of the protein profiles in general. We were able to detect the largest Mahalanobis distance between the cells incubated with POAG serum and an elevated pressure in comparison to control cells incubated with healthy serum (28.1, p-value<0.01). The Mahalanobis distance between cells incubated with POAG serum to healthy controls was 6.5 and between cells incubated with healthy serum+pressure and control cells 4.6 (p- values<0.01). The Mahalanobis distances show the overall changes between experimental groups in comparison to one known/control group.. The larger the distance, the larger the difference between the protein profiles in the experimental groups.

**Figure 3:**
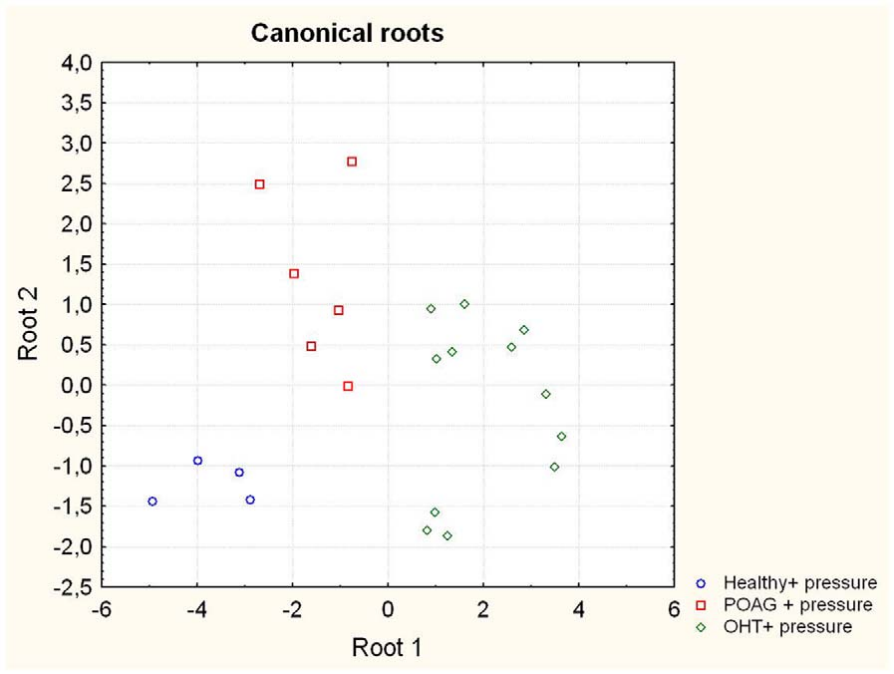
OHT serum also provokes different cell reactions. The graph shows the calculated canonical roots based on the analysis of discriminance of the cells incubated with serum from healthy people, patients suffering from POAG as well as OHT patients. A clear discrimination between the cells on the different experimental groups can be found showing that there are significant differences between the protein profiles and therefore the cell reactions in the different experimental groups.

In the second experiment the analysis showed significant differences between cells incubated with OHT serum and healthy or POAG serum, as well as cells incubated with NTG serum and healthy or POAG serum. Significant differences between protein profiles of cells incubated with NTG or OHT serum were detected. Figure S3 of the supplementary information shows an overview of measured protein profiles of cells incubated with NTG serum in comparison to POAG or healthy serum showing different expression levels of proteins depending on the treatment group. Cells incubated with POAG or healthy serum in comparison to OHT serum also showed significant differences in their protein profiles. Canonical roots were calculated showing a clear differentiation between cells incubated either with POAG, OHT or healthy serum (Figure 3).

### Serum influence's cells most significantly

We detected a 60% loss of viability after stress through an elevated pressure after 48 h (T-test, p<0.01; Table S2). When comparing the viability of the cells incubated either with glaucoma or healthy serum no differences between the experimental groups were detected (T-test, p = 0.94, see Table S3). The calculated variance component analysis demonstrated that the serum had the largest effect on the protein profiles of the cells (59%, Table 1). The pressure only had an 11.6% influence, but had an additional effect of 14% on the serum influence. The large influence of the serum also was detected for the significantly changed proteins, e.g. on the protein at 9192 Da (55.2%, Table 1).

**Table 1:** Variance component analysis.

Relative Variance components (in percent)		
Variable	Influence on general protein profile	Influence on protein 9192 Dalton
**Serum type**	59.1%	55.2%
**Pressure**	11.6%	
**Combination serum type and pressure**	14.0%	9.7%
**Other influences**	15.3%	35.1%

Table 1 shows the calculated relative variance components analysis in percent for both the overall protein profiles of the cells as well as the influence of the different components on one of the significantly differently regulated proteins (9192 Da). The serum type has a large influence on both the overall protein profiles as well as on the single protein. Furthermore an additional effect of serum type and elevated pressure can be detected in both groups.

### Antibodies cause part of effect

After Ab removal the variance component analysis (Table 2) showed an influence of the Abs on the protein profiles of 50.5%. The calculated Mahalanobis distances showed that the overall protein expression profiles of these cells changed significantly when removing the antibodies from the POAG serum (p<0.03; Table 3).

**Table 2:** Variance components analysis after antibody removal.

Relative Variance components after antibody removal (in percent)	
Variable	Influence on general protein profile
**Antibodies**	50.5%
**Serum type**	13.4%
**Other influences**	36.1%

Table 2 shows the calculated relative variance components after antibody removal. We were able to calculate that the antibody removal had a 50% effect on the protein profiles of the cells.

**Table 3:** Mahalanobis distances in comparison to control cells.

Mahalanobis distances to control cells	
Experimental group	Distance
POAG – Antibodies	19,6
POAG	56,1

Table 3 shows the calculated Mahalanobis distances for the cells incubated with POAG serum or POAG serum after antibody removal in comparison to the control cells. The Mahalanaobis distances are a measure to show the similarity or the difference between experimental groups to one defined group, in this case the cells incubated with control serum. The largest Mahalanobis distance can be seen for the protein profiles of the cells incubated with POAG serum. A significant change can be seen for the cells incubated with POAG serum after antibody removal.

To detect whether cells reacted similar to incubation with POAG serum and the Abs of POAG patients, RGC5 cells were incubated with healthy serum, POAG serum and the Abs of POAG patients. A protein profiles analysis was performed. We identified 3124 proteins. 198 Proteins were significantly differently regulated (<−4 or >4 fold different expression) in cells incubated with POAG serum in comparison to cells incubated with healthy serum (The significantly differently regulated proteins are listed in Table S4). 82 Proteins were significantly up- or down- regulated (<−4 or >4 fold different expression) in those cells incubated with the POAG Abs (list of proteins: see Table S5). 56% of the significantly changed proteins in cells incubated with POAG Abs were concordantly regulated in cells incubated with POAG serum. 34% of the proteins significantly changed in cells incubated with POAG Abs showed the same tendency of regulation in cells incubated with POAG serum without being significant in both groups. Only 5% of the significantly changed proteins in cells incubated with POAG Abs showed significant changes in the opposite direction in cells incubated with POAG serum.

The analysis of the changed proteins showed that they mostly were associated with cell regulatory functions (see Table S6). Using Ingenuity pathway analysis (Ingenuity® Systems, Redwood City, California, USA) we found, that many of the significantly changed proteins were associated with cell death pathways, cell growth and proliferation pathways or RNA- posttranscriptional modification pathways.

**Figure 4:**
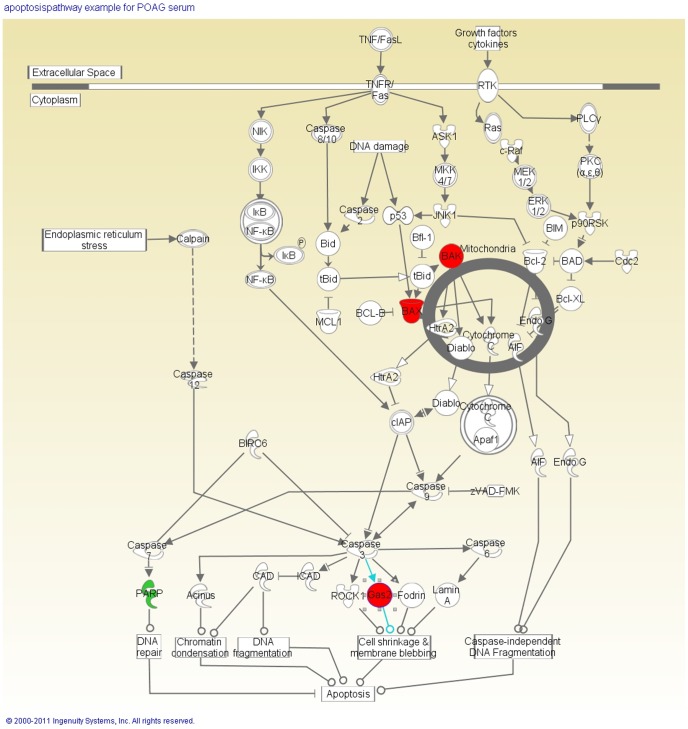
Cell regulatory pathways changed through glaucoma serum. Figure 4 shows changes of proteins belonging to apoptosis pathways in the cells incubated with POAG serum. Red highlighting shows a significant up-regulation of the protein in the cells whereas green highlighting demonstrates a significant down-regulation, both in a pro-apoptotic manner. The pro-apoptotic proteins BAK, BAX and GAS2 were up-regulated whereas the anti-apoptotic protein PARP was significantly down-regulated in the cells incubated with POAG serum. The pathway was built using the Ingenuity pathway analysis software.

Some of the significantly changed proteins were regulated in a pro-apoptotic manner. One of the pathways affected by incubation with POAG serum was a mitochondrial pathway. We found an up-regulation of BAK and BAX both involved in the mitochondrial apoptosis pathway [26] as well as an up-regulation of Gas2 which leads to cell shrinkage and cell blebbing [27]. Furthermore we were able to detect a down-regulation of the anti-apoptotic protein PARP which has a DNA-repair function. The connection between the different proteins is shown in Figure 4. Proteins indicated in a red colour are up-regulated whereas proteins indicated with green are down-regulated.

Proteins differently regulated in the cells incubated with POAG Abs were mostly associated with pathways regulating cellular development and cell cycle. A list of proteins involved in apoptosis and significantly differently regulated in cells incubated with POAG Abs is shown in Table S6.

### Serum effect seems to be dependent on serum concentration

**Figure 5:**
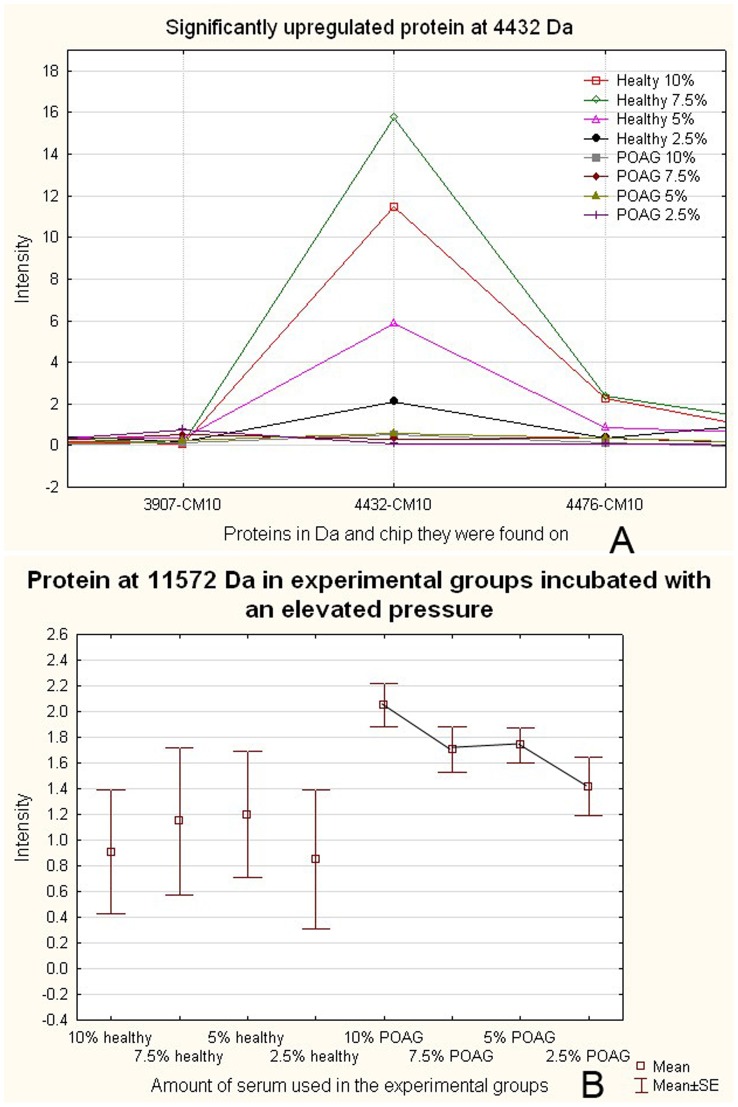
Serum effect is concentration dependant. Figure 5A shows one of the significantly changed proteins in the cells incubated with healthy serum. A concentration dependant behaviour is shown. The cells incbuated with the highest serum levels show the highest protein expression, whereas the cells incubated with the lowest serum concentration show the lowest protein expression. Figure 5B shows a not significantly changed protein either in the cells incubated with POAG or healthy serum. A concentration dependant expression level can be detected in the cells incubated with POAG serum.

We detected that some of the proteins showed a concentration dependent behavior e.g. the significantly up-regulated protein at 4432 Da (Fig. 5A). When looking at other proteins, we detected many showing a concentration dependent behavior, e.g. the protein at 11572 Da (Fig. 5B).

### Discrimination between glaucoma and non-glaucoma possible

Based on the protein profiles of R28 cells incubated with POAG, NTG or healthy serum, artificial networks were calculated to detect the discrimination potential between glaucoma and non-glaucoma. A receiver operating characteristic (ROC) curve was calculated showing, that discrimination between glaucoma and non-glaucoma serum is possible with a sensitivity of 88% and a specificity of 90%. The area under the curve is 0.92.

## Discussion

The first experiments demonstrated in this manuscript focused on the effect of elevated pressure on the cells as well as the effect the serum had on the protein profiles of the cells. We were able to detect a significant loss of viability (60%) of cells incubated with an elevated pressure, correlating with already published data showing a loss of viability of neuronal cells [28] and rgc in vitro under elevated pressure [11]. However, we did not detect significant changes in the viability of cells incubated with glaucoma in comparison to healthy serum. As glaucoma is a slow proceeding disease we believe that significant changes of viability of cells after a short incubation time with patient's serum seems unlikely. However we were able to detect that the serum of glaucoma patients had a very large (59%) and significant influence on the protein profiles of the cells. This influence was potentiated through an elevated pressure (Fig. 2C and 4A). This analysis underlines the hypothesis that the serum of glaucoma patients, by influencing the proteins in the cells, leads to imbalance of proteins in cell regulatory processes and eventually can lead a higher vulnerability of the cells against stress factors such as an elevated pressure.

### Specific changes in the protein profiles depending on serum type

We detected highly complex protein and peptide profiles in all experimental groups, showing significant differences not only in the expression of single proteins, but also in the overall protein profiles of the cells. This effect was demonstrated not only for POAG serum but also in cells incubated with NTG serum or OHT serum. In comparison to serum from healthy people, POAG and NTG serum showed significantly different effects. But also when analyzing the protein expression levels between cells incubated with POAG serum or NTG serum we were able to find significant differences. Cells incubated with OHT serum reacted significantly different than cells incubated with either POAG or healthy serum.

Studies show changes of several antibodies in the serum from glaucoma patients that are significantly correlated to different forms of glaucoma. These include specific antibodies against small heat shock proteins e.g. Hsp 27 or α-crystallins [29], α-fodrin [30], antiphosphatidylserine [31] or γ-enolase [32], but also complex changes in the autoantibody profiles against retinal or other ocular antigens [17]. POAG patients show many autoantibodies against retinal antigens, whereas NTG patients show the largest differences in the autoantibodies against optic nerve antigens [17], [33]. Changes between OHT and POAG patients or healthy people were also observed in other studies [18]. Combining this information with the results shown in this manuscript underlines the theory that the antibodies in the serum of glaucoma patients have a significant influence in the pathogenesis of the disease glaucoma and could be the serum factor leading to changes in cell regulatory processes.

### Significantly changed proteins have regulatory functions

In the first study shown in this manuscript several of the significantly changed proteins were identified. Using the Seldi-TOF-MS approach limits the amount of protein identifications. Using Seldi-TOF MS in combination with the statistical analysis we gained information about the molecular weight of the significantly changed proteins. In order to identify the proteins a SDS-Page had to be performed and the proteins of the bands possibly containing the proteins of interest had to be eluted. These then were measured with Seldi-Tof-MS in order to detect whether the protein of interest was enriched in the fraction. This was the first limiting step of the procedure. If the protein was in the eluted fraction a tryptic digestion was performed and the peptide measured with Maldi-TOF-MS in order to identify the wanted protein. Even after this step the identification of the protein was only secure if the score received from the database was high enough and the measured peptide additionally was a known fragment of the protein. This is a very well-known limitation of top-down Seldi-Tof analysis, which also has been discussed in the literature before [34]. Nevertheless, even those proteins not identified and only known by their molecular weight, can add important knowledge as shown in many proteome publications [35], [36]. Therefore, in our set-up, only a few of the significantly changed proteins were identified. Those proteins securely identified are known to be part of cellular regulatory processes.

We detected changes in the expression level of histone 4 and profilin in cells incubated with POAG serum in comparison to healthy serum. Histones are core proteins of eukaryotic cells and responsible for the spooling of the DNA and the regulation of genes. Histones, can be post-translationally modified e.g. by acetylation or methylation. Changes in the modifications and location of histones have been found in several other neurodegenerative diseases e.g. Alzheimer disease [37], [38], but also changes of the histones of Parkinson disease patients have been detected [39]. Diseases, such as Alzheimer, show overall similarities in terms of apoptosis of neurons [40] as well as autoimmune components [41], [42], but also more specific similarities such as changed beta amyloid and Tau protein levels either in the cerebral fluid of Alzheimer patients [43] or the vitreous of glaucoma patients [44]. Profilin, down-regulated in those cells incubated with POAG serum and an elevated pressure, is an actin binding protein and plays a role in membrane trafficking [45]. Studies show a correlation of Profilin with glaucoma and were able to show a hypotensive effect, when applying Profilin topically [46]. Profilin was shown to be up-regulated in the substantia nigra of Parkinson patients [47]. In contrast we were able to show a down-regulation of Profilin in the cells incubated with glaucoma serum in combination with an elevated pressure. Profilin also has a function in neuronal excitability and plays a role in actin dynamics [45], [48]. Changes in the expression level of Profilin in cells therefore can lead to changes in cell dysfunction as shown in other studies [49]. These results emphasize the hypothesis that the serum, respectively a serum component, of glaucoma patients has an influence on cell regulatory processes possibly leading to a higher vulnerability of the cells towards stress factors. Additionally, as discussed above, the different cell reactions towards the different serum types could be correlated with the different autoantibody levels in the serum depending on the type of glaucoma the patient was suffering from. Therefore further experiments were performed in order to analyze the antibody effect on the cells.

### Antibodies play a role in the serum effect on the cells

After removal of the antibodies from the POAG serum we were able to detect a large influence (50%; Fig. 5A and B) of the Ab removal on the protein profiles of the cells. By removing the antibodies from the serum we could demonstrate that the antibodies seem to be involved in the serum effect of POAG serum on the cells. Using this approach we were not able to show which changes the Abs themselves provoke in the cells. Therefore further experiments were performed incubating the cells also with POAG Abs after their removal from the serum. By performing a capillary LC-ESI-MSMS rather than a Seldi-TOF-MS measurement we also were able to directly identify the proteins. When comparing the significantly changed proteins of cells incubated either with POAG serum or just with POAG Abs we found that only 5% of the proteins were significantly differently regulated, meaning an up-regulation in one group and a down-regulation on the other group in comparison to cells incubated with healthy serum.

In the cells incubated with POAG serum we found, that several of the proteins involved in the apoptosis of cells, e.g. BAX (5 fold up-regulated), BAK1 (22.9 fold up-regulated), PARP1 (8.8 fold down-regulated) and GAS2 (7.8 fold up-regulated), were significantly differently regulated in a pro-apoptotic manner. BAX belongs to the Bcl-2 family and is involved in the intrinsic apoptosis pathway as a pro-apoptotic protein [50].BAK1 also is a pro-apoptotic member of the Bcl-2 family [51]. Up-regulation of these factors can lead to apoptosis of cells. Furthermore the up-regulation of GAS2, a pro-apoptotic protein [27], as well as the down-regulation of PARP1, an enzyme involved in the repair of the DNA [52], can lead to increase of apoptosis of the cells. In previous studies BAX was correlated with neurodegeneration in glaucoma [53].

When analyzing the proteins changed in the cells incubated with just the POAG Abs we were also detect many changes in proteins involved in apoptosis pathways of the cells. Many of the proteins were regulated in a pro-apoptotic manner, e.g. GAS2, which was also found to be regulated in a pro-apoptotic manner in the cells incubated with POAG serum, but we also found proteins regulated in an anti-apoptotic manner. 11 of the significantly differently regulated proteins involved in the apoptosis of cells were changed in a pro-apoptotic manner whereas 8 proteins were changed in an anti-apoptotic manner.BAK1 was found to be down-regulated in the cells incubated with POAG Abs, whereas it was up-regulated in the cells incubated with POAG serum. Looking at the complexity of cell cycle processes it would be surprising to find a concordant regulation of all of the significantly changed proteins involved in cell cycle/apoptosis processes in the different experimental groups.

But we believe that these results underline our first hypothesis that the serum of glaucoma patients can lead to changes in cell regulatory processes. The balance between proteins in charge of cell survival and cell death, e.g. apoptosis, gets disturbed and long-term, possibly in combination with a further stress factor can lead to a predominance of the pro-apoptotic proteins. The anti-apoptotic counterpart proteins eventually can no longer countersteer and cell apoptosis is induced. Furthermore our second hypothesis is underlined by the fact that many of the proteins significantly differently regulated in the cells incubated with POAG serum are found to be regulated in the same way in in cells incubated with POAG Abs. Additionally these cells also show many changes in proteins concerning apoptotic processes.

### Are the protein expression changes specific for rgcs in glaucoma?

When analyzing the cells incubated with POAG or healthy serum we were able to show that the discrimination between glaucoma or non-glaucoma serum was possible with a sensitivity of 88% and a specificity of 90%. In order to answer the question whether these changes are specific for retinal ganglion cells in glaucoma further experiments are needed, as the cells used in these studies (R28 cells or RGC5 cells) both are not pure rgc cultures. R28 cells are known to have properties of other cells such as photoreceptors or glial cells and have to be looked at as neuroretinal cells or retinal precursor cells. [54], [55]. Furthermore the RGC5 cells show properties at least also of neuronal cells and therefore also should be used as neuroretinal cells rather than rgc. Furthermore not all of the RGC5 cells show the rgc specific marker Thy1.2 [23]. In the past these cells have been used in many studies to explore the pathogenesis of glaucoma.

In summary, this study shows significant and specific changes in the way the neuroretinal cells react to serum of glaucoma patients. Significantly differently expressed proteins were detected, that play crucial roles in cellular signaling pathways not only in cells incubated with POAG serum but also in cells only incubated with the Abs of POAG patients. We were able to underline both our hypothesis that the serum of glaucoma patients can lead to changes of protein profiles of retinal cells, which then can lead to a higher vulnerability of the cells toward stress factors, as well as the assumption that the antibodies in the serum of glaucoma patients could be the serum factor in charge of the seen serum effects.

## Supporting Information

Figure S1 **Confirmation of the AB removal after treatment of the POAG serum with Protein G beads.** The antibodies of the POAG serum were removed with Protein G Beads. The AB fraction was eluted from the beads and measured with Seldi-TOF MS. Only if we were able to detect an AB peak in the Seldi-TOF-MS measurements the serum was used as AB free serum. The beads were used in abundance to the serum.
(TIF)

Figure S2 **Confirmation of the AB removal after treatment of the POAG serum with Protein G beads.** Ab removal from POAG serum was performed using Nab Protein G Spin Kit (Thermo scientific). The Ab removal was performed according to the manufacturer's protocol for the amount of 5% (500 µl) serum for each experiment. The Ab's then were used in order to incubate the cells. Beforehand the Kit was performed several times to assure that we were able to show reproducible Ab removal. The bands show different proteolytic components fof serum IgG. The lanes A, B and C show Ab fractions, the 4^th^ lane shows SeeBluePlus marker.
(TIF)

Figure S3 **Significantly changed protein profiles in cells incubated with NTG serum.** Figure S3 shows a pullout of the measured protein profiles including cells incubated with NTG serum. Again complex protein profiles are shown. Changes in the proteins profiles between the experimental groups can be seen. The cells incubated with NTG serum react differently than cells incubated either with POAG serum or healthy serum.
(TIF)

Table S1 **Significantly changed proteins with their p- value and identification.** Table S1 shows the proteins significantly differently regulated in R28 after incubation with healthy or POAG serum und a normal or an elevated pressure of 15000 Pa (112 mmHg). The first column shows the molecular weight of the protein with information of the Seldi chip the protein was measured on. The second column shows the p- Value of the significantly changed protein. In the third column the identified proteins are named.
(DOCX)

Table S2 **Loss of cell viability after incubation with elevated pressure.** The cells were incubated with bovine serum under a normal or an elevated pressure of 112 mmHg. We were able to detect that the cells incubated with bovine serum and an elevated pressure showed a loss of viability of nearly 60%.
(DOCX)

Table S3 **No difference in viability when incubating cells either with POAG or healthy serum.** The viability of the cells incubated either with POAG or healthy serum under a normal or elevated pressure was measured and compared. We were not able to detect a significant difference between the viability of the cells incubated with POAG serum and an elevated pressure or the cells incubated with healthy serum and an elevated pressure. Both groups showed a loss of viability after incubation with an elevated pressure of nearly 62%. The table shows the values measured in the WST-1 test.
(DOCX)

Table S4 **Significantly changed proteins in RGC5 cells after incubation with POAG serum.** We identified 3124 proteins in RGC 5 cells incubated with healthy serum, POAG serum or POAG antibodies. 198 Proteins were significantly differently regulated (<−4 or >4 fold expression) in cells incubated with POAG serum in comparison to cells incubated with healthy serum. The first column shows the short name of the protein and the second column shows the fold change of the protein in the cells incubated wit POAG serum in comparison to healthy serum.
(DOCX)

Table S5 **Significantly changed proteins in cells incubated with POAG Abs in comparison to healthy serum.** We identified 3124 proteins in RGC 5 cells incubated with healthy serum, POAG serum or POAG antibodies. 82 Proteins were significantly up- or down- regulated in those cells incubated with the POAG Abs.
(DOCX)

Table S6 **Significantly changed proteins involved in apoptosis in cells incubated with POAG Abs.** RGC5 cell were incubated with healthy serum, POAG serum or POAG antibodies. Proteins involved in apoptosis regulation were found to be significantly differently regulated in those cells incubated with either POAG serum or POAG Abs. The proteins differently regulated in the cells incubated with POAG Abs are shown in this table. The ID of the protein as well as the Gene information is given. Furthermore in column 3 the prediction whether the protein is regulated in a pro-apoptotic manner (increased) or an anti-apoptotic manner (decreased) is shown. Column 4 shows the fold change of the protein in the cells and the last column shows whether in general the protein is pro-apoptotic (increased) or anti-apoptotic (decreased).
(DOCX)
