# Supplementary figures and images for: Serum and Antibodies of Glaucoma Patients Lead to Changes in the Proteome, Especially Cell Regulatory Proteins, in Retinal Cells
Source: PLoS One. 2012 Oct 11;7(10):e46910. doi: 10.1371/journal.pone.0046910 (PMC3469602; doi:10.1371/journal.pone.0046910)

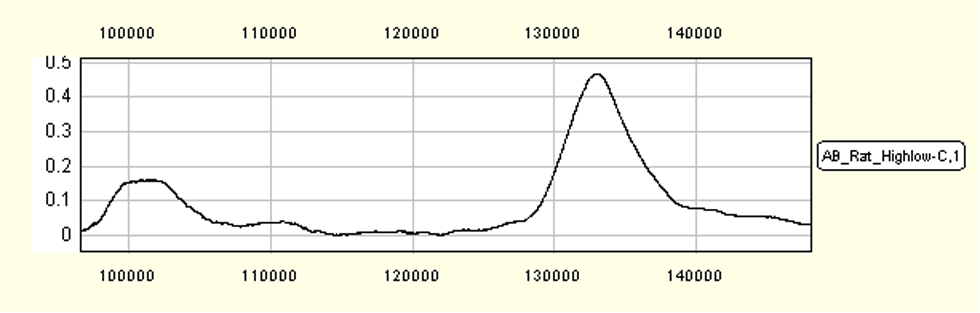

Supplement: Figure S1 — Confirmation of the AB removal after treatment of the POAG serum with Protein G beads. The antibodies of the POAG serum were removed with Protein G Beads. The AB fraction was eluted from the beads and measured with Seldi-TOF MS. Only if we were able to detect an AB peak in the Seldi-TOF-MS measurements the serum was used as AB free serum. The beads were used in abundance to the serum. (TIF) [file pone.0046910.s001.tif]

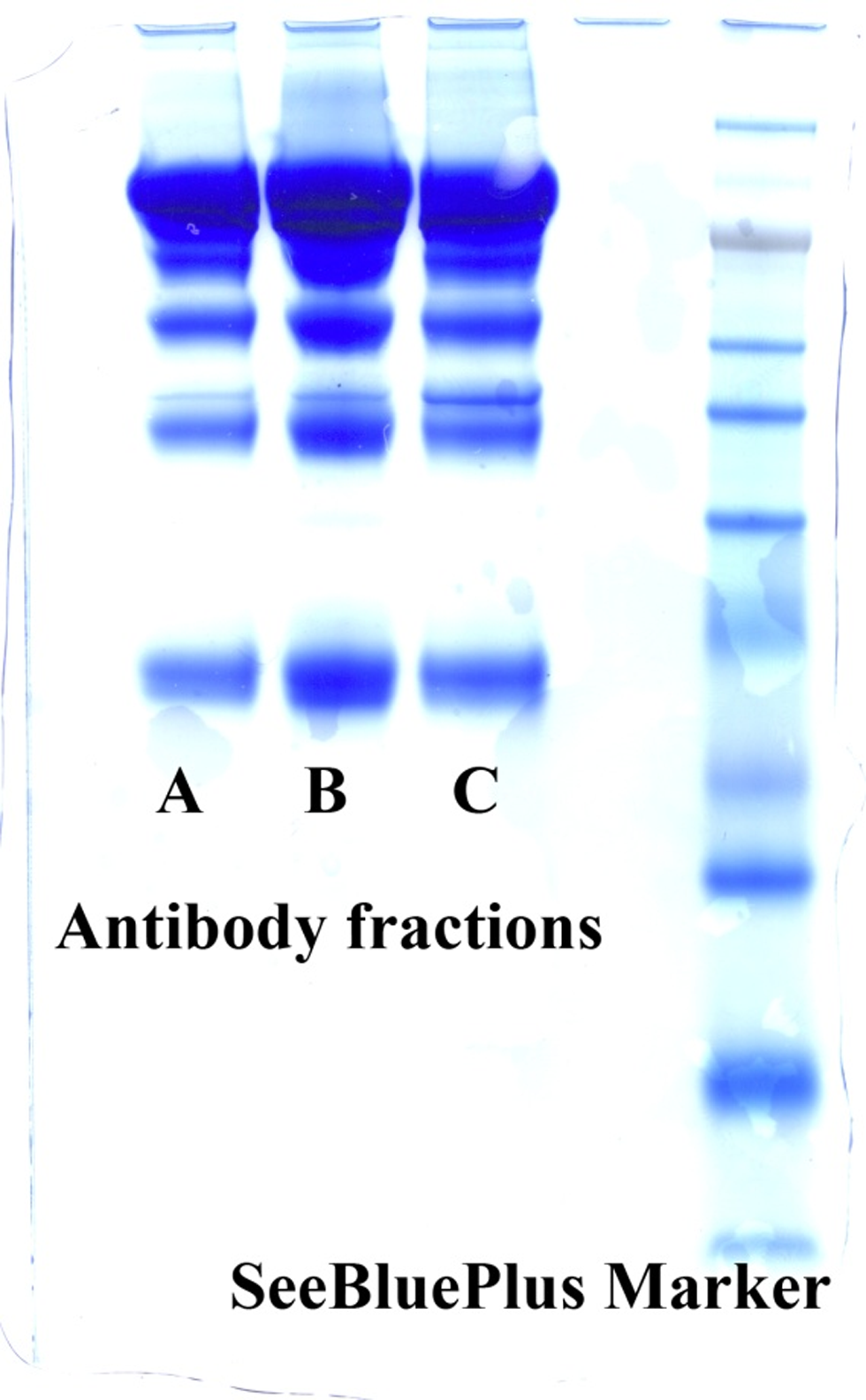

Supplement: Figure S2 — Confirmation of the AB removal after treatment of the POAG serum with Protein G beads. Ab removal from POAG serum was performed using Nab Protein G Spin Kit (Thermo scientific). The Ab removal was performed according to the manufacturer's protocol for the amount of 5% (500 µl) serum for each experiment. The Ab's then were used in order to incubate the cells. Beforehand the Kit was performed several times to assure that we were able to show reproducible Ab removal. The bands show different proteolytic components fof serum IgG. The lanes A, B and C show Ab fractions, the 4th lane shows SeeBluePlus marker. (TIF) [file pone.0046910.s002.tif]

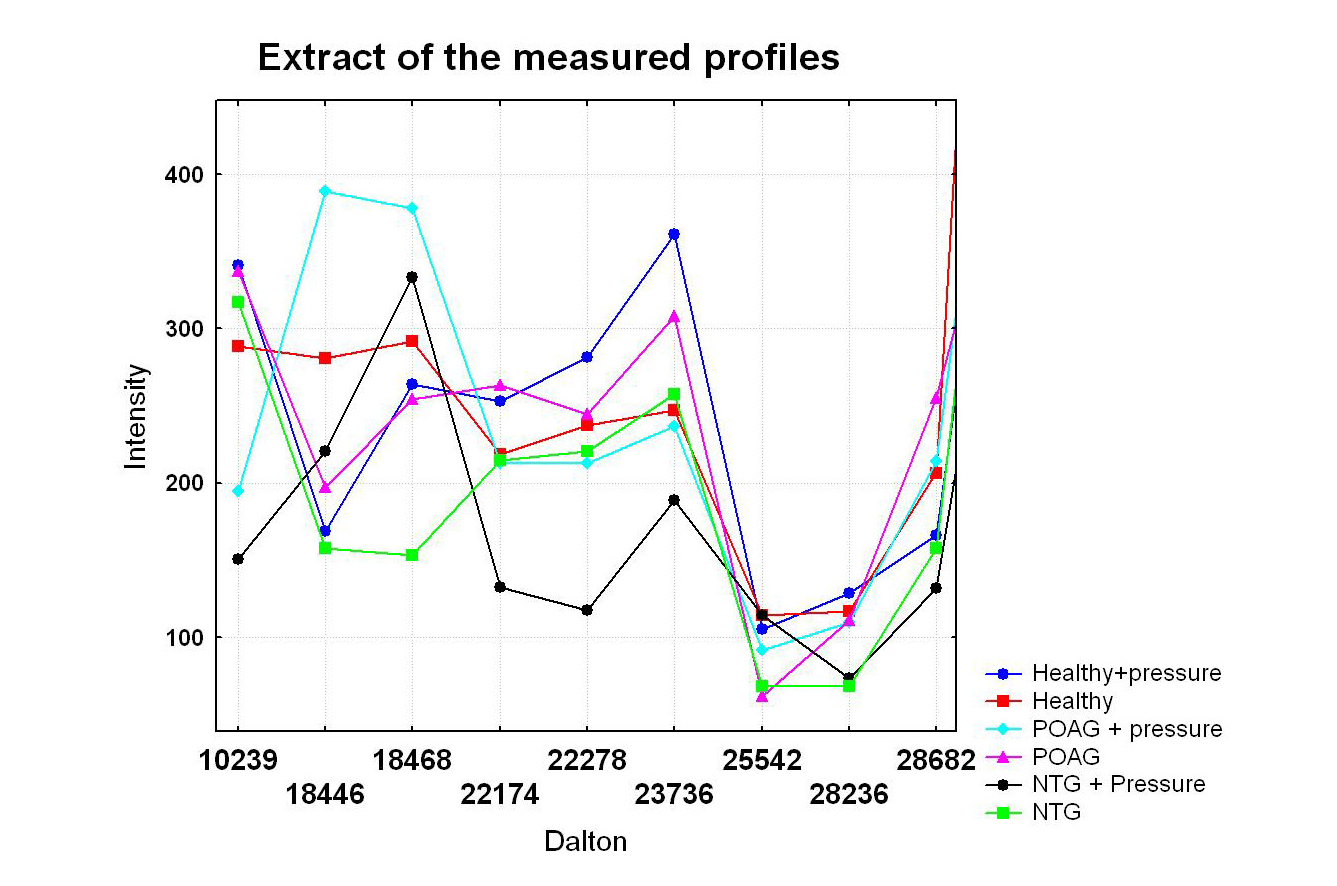

Supplement: Figure S3 — Significantly changed protein profiles in cells incubated with NTG serum. Figure S3 shows a pullout of the measured protein profiles including cells incubated with NTG serum. Again complex protein profiles are shown. Changes in the proteins profiles between the experimental groups can be seen. The cells incubated with NTG serum react differently than cells incubated either with POAG serum or healthy serum. (TIF) [file pone.0046910.s003.tif]
