# Supplementary material for: Serum and Antibodies of Glaucoma Patients Lead to Changes in the Proteome, Especially Cell Regulatory Proteins, in Retinal Cells
Source: PLoS One. 2012 Oct 11;7(10):e46910. doi: 10.1371/journal.pone.0046910 (PMC3469602; doi:10.1371/journal.pone.0046910)
Supplement: Table S1 — Significantly changed proteins with their p- value and identification. Table S1 shows the proteins significantly differently regulated in R28 after incubation with healthy or POAG serum und a normal or an elevated pressure of 15000 Pa (112 mmHg). The first column shows the molecular weight of the protein with information of the Seldi chip the protein was measured on. The second column shows the p- Value of the significantly changed protein. In the third column the identified proteins are named. (DOCX) [file pone.0046910.s004.docx]

Table S1: Significantly changed proteins with their p- value and identification

| Molecular weight of significantly changes protein with chip information | p-value | Identification |
| --- | --- | --- |
| 12134-H50 | <0.001 |  |
| 4678-H50 | <0.001 |  |
| 4612-H50 | <0.001 |  |
| 9177-CM10 | <0.001 |  |
| 9192-H50 | <0.001 | Histone H4 |
| 12147-CM10 | <0.001 |  |
| 12390-H50 | <0.001 | Profilin |
| 9355-H50 | <0.001 |  |
| 4604-CM10 | <0.001 |  |
| 9253-H50 | <0.001 |  |
