# Supplementary material for: Serum and Antibodies of Glaucoma Patients Lead to Changes in the Proteome, Especially Cell Regulatory Proteins, in Retinal Cells
Source: PLoS One. 2012 Oct 11;7(10):e46910. doi: 10.1371/journal.pone.0046910 (PMC3469602; doi:10.1371/journal.pone.0046910)
Supplement: Table S2 — Loss of cell viability after incubation with elevated pressure. The cells were incubated with bovine serum under a normal or an elevated pressure of 112 mmHg. We were able to detect that the cells incubated with bovine serum and an elevated pressure showed a loss of viability of nearly 60%. (DOCX) [file pone.0046910.s005.docx]

Table S2: Loss of cell viability after incubation with elevated pressure

| Viability (%) of the cells after incubation with an elevated pressure vs. non elevated pressure | |
| --- | --- |
| Sample911a | 50.2 |
| Sample911b | 45.1 |
| Sample911c | 47.3 |
| Sample911d | 39.5 |
| Sample911e | 39.2 |
| Sample13031a | 47.3 |
| Sample13031b | 46.2 |
| Sample13031c | 34.5 |
| Sample13031d | 34.4 |
| Sample13031e | 34.0 |
| Sample13032a | 49.8 |
| Sample13032b | 39.5 |
| Sample13032c | 34.6 |
| Sample13032d | 38.8 |
| Sample13032e | 33.0 |
| Sample13033a | 67.5 |
| Sample13033b | 44.8 |
| Sample13033c | 31.0 |
| Sample13033d | 34.7 |
| Sample13033e | 39.7 |
| Standard deviation | 8.3 |
| Mean value | 41.6 |
