# Supplementary material for: Serum and Antibodies of Glaucoma Patients Lead to Changes in the Proteome, Especially Cell Regulatory Proteins, in Retinal Cells
Source: PLoS One. 2012 Oct 11;7(10):e46910. doi: 10.1371/journal.pone.0046910 (PMC3469602; doi:10.1371/journal.pone.0046910)
Supplement: Table S3 — No difference in viability when incubating cells either with POAG or healthy serum. The viability of the cells incubated either with POAG or healthy serum under a normal or elevated pressure was measured and compared. We were not able to detect a significant difference between the viability of the cells incubated with POAG serum and an elevated pressure or the cells incubated with healthy serum and an elevated pressure. Both groups showed a loss of viability after incubation with an elevated pressure of nearly 62%. The table shows the values measured in the WST-1 test. (DOCX) [file pone.0046910.s006.docx]

Table S3: No difference in viability when incubating cells either with POAG or healthy serum.

| Sample  (each sample is the mean of 2x10 single meassurements) | WST- Test cells with healthy serum without an elevated pressure | WST- Test cells with healthy serum and an elevated pressure | WST- Test cells with POAG serum without an elevated pressure | WST- Test cells with POAG serum and an elevated pressure |
| --- | --- | --- | --- | --- |
| Sample 911a | 0,20 | 0,09 | 0,18 | 0,07 |
| Sample 911b | 0,19 | 0,09 | 0,19 | 0,08 |
| Sample 13011a | 0,27 | 0,09 | 0,20 | 0,10 |
| Sample 13011b | 0,27 | 0,09 | 0,28 | 0,09 |
| Sample 13012a | 0,21 | 0,08 | 0,23 | 0,09 |
| Sample 13012b | 0,21 | 0,07 | 0,25 | 0,09 |
| Sample 13013a | 0,26 | 0,09 | 0,22 | 0,10 |
| Sample 13013b | 0,22 | 0,09 | 0,28 | 0,09 |
| Sample 0202a | 0,24 | 0,09 | 0,24 | 0,08 |
| Standard deviation | 0,03 | 0,01 | 0,03 | 0,01 |
| Cells surviving the elevated pressure in % |  | **38,2764175** |  | **38,4443069** |
