# Supplementary material for: Serum and Antibodies of Glaucoma Patients Lead to Changes in the Proteome, Especially Cell Regulatory Proteins, in Retinal Cells
Source: PLoS One. 2012 Oct 11;7(10):e46910. doi: 10.1371/journal.pone.0046910 (PMC3469602; doi:10.1371/journal.pone.0046910)
Supplement: Table S4 — Significantly changed proteins in RGC5 cells after incubation with POAG serum. We identified 3124 proteins in RGC 5 cells incubated with healthy serum, POAG serum or POAG antibodies. 198 Proteins were significantly differently regulated (<−4 or >4 fold expression) in cells incubated with POAG serum in comparison to cells incubated with healthy serum. The first column shows the short name of the protein and the second column shows the fold change of the protein in the cells incubated wit POAG serum in comparison to healthy serum. (DOCX) [file pone.0046910.s007.docx]

Table S4: Significantly changed proteins in RGC5 cells after incubation with POAG serum

| ShortName | Fold Change in RGC5 cells incubated with POAG serum (rounded to two decimal places) |
| --- | --- |
| O75396 | -12,38 |
| O89086 | -7,01 |
| P15532 | -4,55 |
| P16045 | -4,68 |
| P35276 | 5,37 |
| P60059 | 22,19 |
| P62823 | 5,37 |
| P63011 | 5,37 |
| P84090 | -7,61 |
| Q7L9L4 | 5,52 |
| Q8BHC1 | 5,37 |
| Q8BHD0 | 5,37 |
| Q8K386 | 6,65 |
| Q9CPQ3 | 4,31 |
| Q9CQ19 | -4,32 |
| Q9CQV6 | 8,88 |
| A2A9T0 | 6,81 |
| A2ABV5 | -4,1 |
| A6H6E9 | -8,00 |
| B1AXP6 | -4,06 |
| B2RXC1 | -5,00 |
| B2RY56 | -4,18 |
| O08734 | 5,02 |
| O08756 | 6,63 |
| O08997 | -7,52 |
| O35250 | -12,27 |
| O35593 | 4,46 |
| O35685 | -5,32 |
| O35900 | -5,22 |
| O55189 | 5,27 |
| O70372 | -4,45 |
| O70491 | -5,20 |
| O88544 | -4,26 |
| P00015 | -4,02 |
| P06240 | -5,20 |
| P07091 | -4,50 |
| P0C027 | -4,12 |
| P10923 | -25,66 |
| P11103 | -8,81 |
| P11862 | 7,87 |
| P12382 | 9,84 |
| P18155 | -4,39 |
| P20352 | -6,84 |
| P23242 | -5,43 |
| P23492 | 804,59 |
| P26516 | 7,65 |
| P27512 | -7,63 |
| P28352 | 476,36 |
| P30416 | -8,42 |
| P46062 | -31,10 |
| P46664 | 4,32 |
| P47856 | -4,26 |
| P49290 | -4,11 |
| P50284 | 13,54 |
| P51807 | -4,98 |
| P51855 | -4,04 |
| P53368 | 13,06 |
| P56394 | 15,91 |
| P56959 | -7,29 |
| P58059 | 5,50 |
| P58321 | 6,54 |
| P58681 | -5,44 |
| P60521 | -6,41 |
| P60766 | 6,84 |
| P61161 | 4,28 |
| P61164 | 6,02 |
| P62313 | -4,66 |
| P63056 | -4,66 |
| P70175 | -6,18 |
| P70303 | 12,92 |
| P70338 | -4,11 |
| P70425 | -5,60 |
| P70699 | 10,91 |
| P80560 | -4,01 |
| P83917 | 197,46 |
| P83940 | -4,36 |
| P97351 | -18,37 |
| Q07813 | 22,96 |
| Q3SXD3 | 10,77 |
| Q3TPE9 | -4,17 |
| Q3TW96 | 15,04 |
| Q3U1J4 | -4,13 |
| Q3UHX2 | -5,29 |
| Q3UIW5 | -201,00 |
| Q3UMW8 | -140,04 |
| Q3UZP0 | 173,47 |
| Q497K7 | -8,62 |
| Q505K2 | -6,11 |
| Q5SVL6 | -7,34 |
| Q60931 | -4,06 |
| Q61548 | -4,50 |
| Q61554 | 4,86 |
| Q61647 | 9,26 |
| Q61792 | -6,44 |
| Q62288 | 6,45 |
| Q62422 | -4,44 |
| Q64704 | -4,18 |
| Q6NWV3 | -4,84 |
| Q6P8I4 | -7,08 |
| Q6P9P0 | -5,42 |
| Q6P9Q6 | -5,74 |
| Q6R891 | -4,27 |
| Q6TEK5 | 24,11 |
| Q6V595 | -4,57 |
| Q6ZPQ6 | -6,00 |
| Q71LX4 | -4,44 |
| Q7TSL0 | -5,65 |
| Q80TS7 | -4,27 |
| Q80U12 | 4,05 |
| Q80VF6 | -94,84 |
| Q80WR1 | 4,24 |
| Q8BGY3 | -5,97 |
| Q8BHA1 | -4,31 |
| Q8BHC4 | -8,17 |
| Q8BI73 | -4,90 |
| Q8BJQ9 | -7,54 |
| Q8BK64 | -7,32 |
| Q8BVG4 | -6,60 |
| Q8BVQ5 | -8,41 |
| Q8BWZ3 | -8,49 |
| Q8BXX9 | -5,23 |
| Q8C1F4 | -7,53 |
| Q8CDN6 | -6,17 |
| Q8CGY8 | 5,77 |
| Q8JZN5 | -4,42 |
| Q8K0C4 | 19,08 |
| Q8K0Z9 | -6,12 |
| Q8K2V1 | 6,74 |
| Q8R0F6 | -4,15 |
| Q8R1Q0 | -5,18 |
| Q8R2R9 | 6,41 |
| Q8R3N6 | -6,55 |
| Q8R409 | -4,75 |
| Q8VCH0 | 5,56 |
| Q8VDP6 | 237,66 |
| Q8VEB1 | -4,99 |
| Q8VEM8 | -9,50 |
| Q8VI75 | 32,02 |
| Q91V51 | -4,35 |
| Q91VR7 | 8,88 |
| Q91WK2 | 18,71 |
| Q91YP0 | -4,21 |
| Q920A5 | 6,79 |
| Q920E5 | 5,83 |
| Q921F2 | 6,98 |
| Q924T2 | 9,47 |
| Q925K9 | 5,61 |
| Q99MD9 | -4,61 |
| Q99MT6 | -4,51 |
| Q99NA9 | -7,12 |
| Q99PG0 | -5,04 |
| Q9CPR8 | -6,52 |
| Q9CQ60 | -4,37 |
| Q9CQ80 | 18,83 |
| Q9CQA1 | -4,30 |
| Q9CQH3 | -4,49 |
| Q9CQH8 | 36,50 |
| Q9CQS8 | -7,44 |
| Q9CR61 | 5,70 |
| Q9CR67 | -4,26 |
| Q9CRC0 | -4,38 |
| Q9CX56 | -6,40 |
| Q9CXY6 | 6,61 |
| Q9CY50 | 23,70 |
| Q9CZX0 | -5,27 |
| Q9D0T1 | -7,15 |
| Q9D154 | 9,33 |
| Q9D1H7 | 19,12 |
| Q9D1R9 | -16,04 |
| Q9D6Z1 | -5,11 |
| Q9D710 | 78,90 |
| Q9D855 | -5,10 |
| Q9DAR7 | 7,49 |
| Q9DB05 | 5,20 |
| Q9DCT1 | 12,31 |
| Q9DCT8 | -4,15 |
| Q9DCZ1 | -13,39 |
| Q9ERF3 | 25,42 |
| Q9ERH6 | -5,04 |
| Q9ES97 | -4,68 |
| Q9ESF1 | 11,06 |
| Q9JHI5 | -4,27 |
| Q9JHU3 | -5,77 |
| Q9JI46 | -4,12 |
| Q9JJF3 | -5,63 |
| Q9JJW5 | -4,69 |
| Q9JJW6 | -4,61 |
| Q9JKV2 | -4,53 |
| Q9JLI6 | -7,32 |
| Q9JM52 | 4,81 |
| Q9JM55 | -6,16 |
| Q9JMA1 | -4,88 |
| Q9JMB8 | 7,45 |
| Q9JME7 | 14,80 |
| Q9QUR7 | -6,97 |
| Q9QXT6 | 59,37 |
| Q9Z0H8 | 6,18 |
