# Supplementary material for: Serum and Antibodies of Glaucoma Patients Lead to Changes in the Proteome, Especially Cell Regulatory Proteins, in Retinal Cells
Source: PLoS One. 2012 Oct 11;7(10):e46910. doi: 10.1371/journal.pone.0046910 (PMC3469602; doi:10.1371/journal.pone.0046910)
Supplement: Table S5 — Significantly changed proteins in cells incubated with POAG Abs in comparison to healthy serum. We identified 3124 proteins in RGC 5 cells incubated with healthy serum, POAG serum or POAG antibodies. 82 Proteins were significantly up- or down- regulated in those cells incubated with the POAG Abs. (DOCX) [file pone.0046910.s008.docx]

Table S5: Significantly changed proteins in cells incubated with POAG Abs in comparison to healthy serum.

| Short name of protein | Fold Change protein in cells incubated with POAG Ab‘s  (rounded to two decimal places) |
| --- | --- |
| O54891 | 6,53 |
| O75396 | -8,18 |
| P15532 | -4,02 |
| P60059 | -9,35 |
| P62847 | -5,25 |
| Q8K386 | 4,28 |
| Q8K419 | 6,53 |
| Q9CQV6 | 10,76 |
| A6H6E9 | -7,51 |
| O08734 | -7,36 |
| O54990 | -6,89 |
| O88693 | 4,112 |
| P10923 | -6,44 |
| P11862 | 21,52 |
| P23492 | 18,14 |
| P27048 | -4,60 |
| P27512 | -4,27 |
| P28352 | 9,98 |
| P48754 | -4,26 |
| P53368 | 11,14 |
| P56376 | -4,19 |
| P56394 | 15,71 |
| P58059 | 5,89 |
| P58321 | -29,87 |
| P58467 | 6,53 |
| P60521 | -4,44 |
| P62313 | -4,37 |
| P63163 | -4,60 |
| P70303 | 4,64 |
| P70670 | -4,90 |
| P83917 | 7,43 |
| P97351 | -8,80 |
| P97384 | -5,98 |
| Q09143 | -5,63 |
| Q3SXD3 | 8,77 |
| Q3UMW8 | -5,99 |
| Q3UU94 | 4,15 |
| Q3UZP0 | 8,15 |
| Q497K7 | -5,86 |
| Q5SSH8 | 4,15 |
| Q5SVL6 | -8,26 |
| Q61554 | 5,62 |
| Q61647 | 7,12 |
| Q61790 | -4,84 |
| Q61792 | -5,73 |
| Q64008 | -5,06 |
| Q64701 | -4,23 |
| Q6V595 | -5,84 |
| Q7TSL0 | 12,24 |
| Q80VF6 | -50,02 |
| Q80XU8 | -13,15 |
| Q8BH59 | -4,21 |
| Q8BJQ9 | -5,58 |
| Q8BVQ5 | -10,26 |
| Q8BWZ3 | -5,52 |
| Q8C1F4 | -5,58 |
| Q8CDN6 | -7,64 |
| Q8K0Z9 | -4,31 |
| Q8K3A6 | 4,43 |
| Q8R2R9 | 5,12 |
| Q8VEM8 | -9,06 |
| Q91VR7 | 10,76 |
| Q91WK2 | 7,34 |
| Q99JX4 | -4,39 |
| Q99KI0 | -5,05 |
| Q99MT6 | 8,42 |
| Q99PG0 | -5,00 |
| Q9CQU0 | 4,63 |
| Q9CXE7 | 4,29 |
| Q9CY50 | 20,90 |
| Q9D1M0 | 4,23 |
| Q9D236 | -6,85 |
| Q9DBC7 | 4,59 |
| Q9DCM0 | -4,55 |
| Q9ESF1 | 6,50 |
| Q9JHU3 | -4,55 |
| Q9JJW6 | -4,47 |
| Q9JMB8 | 5,55 |
| Q9QUR7 | -8,76 |
| Q9QXT6 | 5,57 |
| Q9QZM0 | -4,55 |
| Q9R0N0 | 4,22 |
