# Supplementary material for: Serum and Antibodies of Glaucoma Patients Lead to Changes in the Proteome, Especially Cell Regulatory Proteins, in Retinal Cells
Source: PLoS One. 2012 Oct 11;7(10):e46910. doi: 10.1371/journal.pone.0046910 (PMC3469602; doi:10.1371/journal.pone.0046910)
Supplement: Table S6 — Significantly changed proteins involved in apoptosis in cells incubated with POAG Abs. RGC5 cell were incubated with healthy serum, POAG serum or POAG antibodies. Proteins involved in apoptosis regulation were found to be significantly differently regulated in those cells incubated with either POAG serum or POAG Abs. The proteins differently regulated in the cells incubated with POAG Abs are shown in this table. The ID of the protein as well as the Gene information is given. Furthermore in column 3 the prediction whether the protein is regulated in a pro-apoptotic manner (increased) or an anti-apoptotic manner (decreased) is shown. Column 4 shows the fold change of the protein in the cells and the last column shows whether in general the protein is pro-apoptotic (increased) or anti-apoptotic (decreased). (DOCX) [file pone.0046910.s009.docx]

Table S6: Significantly changed proteins involved in apoptosis in cells incubated with POAG Abs

| ID | Genes in Datasheet | Predicition of apoptosis | Fold change  (rounded to two decimal places) | Function of protein in regard to apoptosis |
| --- | --- | --- | --- | --- |
| P11862 | GAS2 (includes EG:14453) | Increased | 21.52 | Increases |
| P23492 | PNP | Decreased | 18.14 | Decreases |
| Q7TSL0 | IFNK | Increased | 12.24 | Increases |
| Q91VR7 | MAP1LC3A | Increased | 10.76 | Increases |
| P28352 | APEX1 | Increased | 9.98 | Increases |
| Q91WK2 | EIF3H | Decreased | 7.34 | Decreases |
| Q8K419 | LGALS4 | Increased | 6.53 | Increases |
| Q61554 | FBN1 | Decreased | 5.62 | Decreases |
| Q9DBC7 | PRKAR1A | Increased | 4.59 | Increases |
| O88693 | UGCG | Decreased | 4.11 | Decreases |
| P15532 | NME1 (includes EG:18102) |  | -4.02 | Affects |
| Q64701 | RBL1 | Increased | -4.23 | Decreases |
| P48754 | BRCA1 | Decreased | -4.26 | Increases |
| P27512 | CD40 | Decreased | -4.27 | Increases |
| Q9DCM0 | ETHE1 | Increased | -4.55 | Decreases |
| Q61790 | LAG3 | Increased | -4.84 | Decreases |
| P97384 | ANXA11 |  | -5.98 | Affects |
| P10923 | SPP1 (includes EG:20750) | Increased | -6.44 | Decreases |
| O08734 | BAK1 | Decreased | -7.36 | Increases |
| Q9QUR7 | PIN1 | Decreased | -8.76 | Increases |
| P97351 | RPS3A |  | -8.80 | Affects |
| P60059 | SEC61G | Increased | -9.35 | Decreases |
